# Supplementary material for: Prediction of Efficacy for Atezolizumab/Bevacizumab in Unresectable Hepatocellular Carcinoma with Hepatobiliary-Phase Gadolinium Ethoxybenzyl-Diethylenetriaminepentaacetic Acid MRI
Source: Cancers (Basel). 2024 Jun 19;16(12):2275. doi: 10.3390/cancers16122275 (PMC11202233; doi:10.3390/cancers16122275)
Supplement: Supplementary file 1 [file cancers-16-02275-s001.zip › cancers-3028573-supplementary.pdf]

Table S1. Pretreatment Gd-EOB-MRI.

INSTITUTION	MODEL (T)	TIMING OF HBP (MINUTES)	REPETITION TIME (MS)	ECHO TIME (MS)	DISPLAY FIELD OF VIEW (MM×MM)	MATRIX SIZE	FLIP ANGLE (DEGREE)	SLICE THICKNESS (MM)
1	3.0	15	3.38–4.70	1.29–1.79	280×298–400×426	384×180–512×280	12/14	1.5
2	1.5	15	3.74	1.45	782×320	288×179	12	2.2
2	3.0	15	3.11	1.13	758×310	288×174	12	1.5
3	3.0	20	3.13	1.18	574×263	320×180	10	4.0
4	3.0	20	2.91–3.45	1.43–1.70	782×320–929×380	320×241–368×295	15	4.0
5	3.0	20	3.46–3.47	1.68–1.69	880×360	320×251	10	2.5
6	1.5	20	3.84–3.90	1.91–1.95	856×350–929×380	256×179–288×201	12	2.0
6	3.0	20	3.64–3.99	1.78–2.00	733×300–929×380	224×257–288×202	10/12	2.0

HBP, hepatobiliary phase
Institution number
1. Nara Medical University
2. Nara Prefecture General Medical Center
3. Nara Prefecture Seiwa Medical Center
4. Kobe University
5. Osaka Metropolitan University
6. Osaka Red Cross Hospital

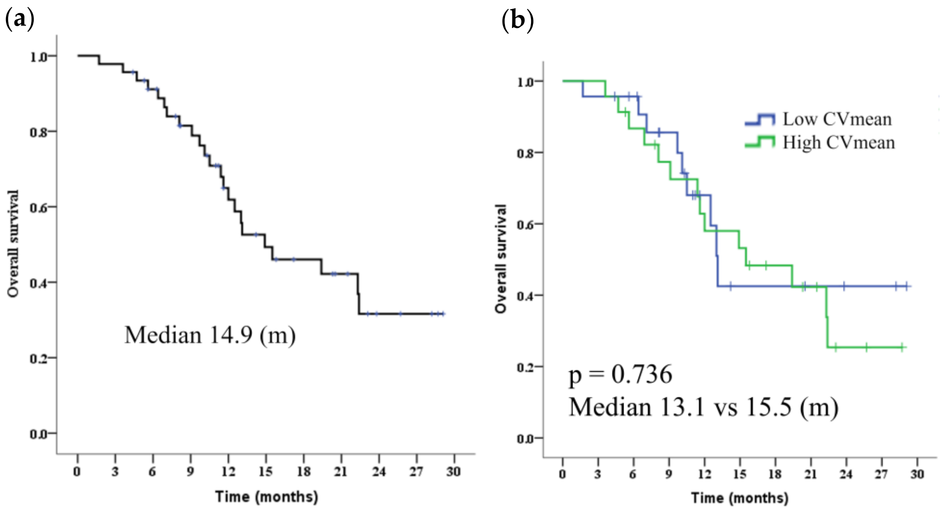

Figure S1. Kaplan–Meier analysis of OS for u-HCC with AB therapy in all patients and comparison between high- and low-CV. (**a**) The median of OS was 14.9 months. (**b**) The median OS was 13.1 and 15.5 months (p = 0.736) in low- and high-CV, respectively. OS, overall survival; AB, atezolizumab and bevacizumab; u-HCC, unresectable hepatocellular carcinoma.
